# Entropy-Based Structural Health Monitoring System for Damage Detection in Multi-Bay Three-Dimensional Structures

**DOI:** 10.3390/e20010049

**Published:** 2018-01-11

**Authors:** Tzu-Kang Lin, Ana Gabriela Laínez

**Affiliations:** Department of Civil Engineering, National Chiao Tung University, Hsinchu 30010, Taiwan

**Keywords:** multi-bay, three-dimensional, structural health monitoring, multi-scale, cross-sample entropy

## Abstract

In this paper, a structural health monitoring (SHM) system based on multi-scale cross-sample entropy (MSCE) is proposed for detecting damage locations in multi-bay three-dimensional structures. The location of damage is evaluated for each bay through MSCE analysis by examining the degree of dissimilarity between the response signals of vertically-adjacent floors. Subsequently, the results are quantified using the damage index (DI). The performance of the proposed SHM system was determined in this study by performing a finite element analysis of a multi-bay seven-story structure. The derived results revealed that the SHM system successfully detected the damaged floors and their respective directions for several cases. The proposed system provides a preliminary assessment of which bay has been more severely affected. Thus, the effectiveness and high potential of the SHM system for locating damage in large and complex structures rapidly and at low cost are demonstrated.

## 1. Introduction

Structural health monitoring (SHM) has attracted considerable attention among engineers, because structures are inevitably subject to internal or external factors that affect their service lives. SHM enables early detection of any damage caused by factors such as the environment or faulty construction, thus facilitating timely maintenance or repair jobs. Over the past two decades, signal-processing techniques have mainly been applied in SHM methods to analyze the measured displacement, velocity or acceleration signals of structures to obtain dynamic characteristics such as basic vibration frequency (natural frequency) and damping. These characteristics contribute to the diagnosis of damage, as well as its condition and possible location [1,2].

In 1999, Wahab and De Roeck [3] used the change in dynamic parameters between the undamaged and damaged conditions of simply-supported and continuous beams to localize damage. In 2000, Maeck et al. [4] identified the degree and location of damage in reinforced concrete beams according to their modal characteristics for dynamic stiffness analysis. Subsequently, vibration-based SHM algorithms and their application limitations were examined and summarized by Chang et al. [5]. The feasibility of using measured modal parameters for damage detection of steel towers through Bayesian probability theory was examined by Lam and Yang [6]. Furthermore, Chen et al. [7] identified bridge bearing damage by using beam modal information as the input to a neural network.

German physicist Clausius [8] first introduced the concept of entropy in 1865 to evaluate the uncertainty of events in a thermodynamic system. Decades later, Shannon [9] proposed the influential Shannon entropy in the field of information theory. Moreover, Kolmogorov [10] defined the notion of entropy for a new class of dynamical systems; Sinai [11] then introduced a definition of entropy that can be applied to all dynamical systems. Jointly, the entropy defined in these two studies was called KS entropy, which was used to measure the complexity of measured time series in D-dimensional dynamic systems. Thereafter, research demonstrated that although KS entropy can be effectively applied to low-dimensional chaotic systems, it cannot be applied to experimental data. The calculation results are affected by various levels of noise that may be involved [12].

In 1991, Pincus [13] developed an analytical method called “approximate entropy” (ApEn) by modifying the KS entropy formula. This method permitted the quantification of regularity in a time series as a single number. The performance of ApEn was tested using clinical data, and the test results demonstrated the predictive and diagnostic capabilities of ApEn. An and Ou [14] proposed the mean curvature difference method based on the ApEn theory and successfully located the damage in the shear frame structures. A modification of ApEn, called “sample entropy” (SampEn), was proposed by Richman and Moorman [15] in 2000. The advantage of SampEn is that the entropy value obtained is not affected by the length of the time series. Moreover, higher relative consistency can be achieved under different parameters such as the threshold *r*, sample length *m* and signal length *N*. Richman and Moorman also argued that comparing a dataset with itself is meaningless; therefore, SampEn does not count self-matches and is free of any bias caused in the entropy estimation.

In 2002, multi-scale entropy (MSE) was proposed by Costa et al. [16,17] to measure the entropy in physiological time series. Traditional entropy-based algorithms that use only a single time scale for analysis may yield misleading results; therefore, a coarse-graining procedure was proposed to obtain more accurate results during entropy calculation. The MSE method was verified by applying it to heartbeat time series of healthy subjects, subjects with congestive heart failure and subjects with atrial fibrillation. The results showed that on multiple scales, healthy heartbeats had the highest entropy values; thus, diagnosing patients through the MSE method is feasible. MSE analysis has been used not only in the medical field, but also in the area of mechanical engineering. The damage condition of roller bearings has been successfully diagnosed through the MSE analysis of vibration signals [18,19].

Similar to ApEn, Cross-ApEn was introduced by Pincus and Singer [20] to analyze the degree of asynchrony of two related time series. Subsequently, Richman and Moorman [15] proposed Cross-SampEn as a superior alternative for the same purpose, because unmatched templates result in undefined probabilities in Cross-ApEn. The performance of both methods was tested using cardiovascular time series, demonstrating that the results obtained using Cross-SampEn had a higher level of relative consistency than those obtained using Cross-ApEn.

In 2013, Fabris et al. [21] applied the SampEn and Cross-SampEn algorithms to electroglottogram and microphone signals. The healthy patients and those with throat or vocal disorders can be identified by quantifying the degree of asynchrony between time series. Subsequently, SHM systems based on the MSE and MSCE algorithms introduced in [15,16,17] in the field of physiology have been proposed to identify damage locations and directions on a single-bay structure [22,23]. The vertical MSCE analysis was performed to identify the damaged floor, and planar MSCE analysis was performed to identify the damage directions. The resulting MSCE curves indicated that a higher degree of synchronicity between two signals yields lower entropy values. Furthermore, time series with high complexity have high entropy values, indicating damage. Considering the higher entropy values obtained for damaged floors, vertical and planar damage index (DI) values were proposed for efficiently quantifying damage. A comparison of healthy and damaged signals revealed positive DI values for damaged floors, whereas negative DI values were observed for healthy floors. Moreover, the parameters of sample entropy and wavelet transformation were optimized to detect the possible crack on a cantilever beam [24], and a cross-entropy optimization technique was also applied to identify the damage of the shear structure component [25].

The aim of the present study was to implement the vertical MSCE analysis coupled with the vertical DI on a large and complex three-bay bi-axial numerical model to identify the damaged floors and damaged bays of the structure. The remainder of this paper is organized as follows: The proposed SHM system is described in Section 2. In Section 3, a numerical evaluation of a three-bay, seven-story steel structure is presented. On the basis of the numerical results, the performance of the MSCE and DI analyses is discussed in Section 4. Finally, Section 5 provides the conclusions.

## 2. SHM Algorithm

### 2.1. SampEn

In this section, SampEn is first introduced to understand more clearly the methods used for the SHM system [13]. As a statistical method for analyzing time series, SampEn estimates the entropy value of a measured time series to quantify the complexity of a system. The results of SampEn, an unbiased refinement of ApEn, are not affected by the time series length or calculation parameters.

For a time series $\left\{X_{i}\right\}=\left\{x_{1},\cdots ,x_{i},x_{N}\right\}$ with length *N*, a vector of *m* data points $u_{m}\left(i\right)=\left\{x_{i},x_{i+1},\cdots ,x_{i+m-1}\right\},1\leq i\leq N-m+1$ can be defined as the template. The combination of all templates with length *m* is represented by the template space *T* of the signal; for example, $\left[x_{i},x_{i+1},\cdots ,x_{i+m-1}\right]$ represents the *i*-th template of the time series. Various N−m+1 templates may constitute the time series. The template space *T*, which is the combination of all N−m+1 templates with length *m*, is expressed as follows:

(1) $$T=\left[\begin{array}{cccc} x_{1} & x_{2} & \cdots & x_{m}\\ x_{2} & x_{3} & \cdots & x_{m-1}\\ \vdots & \vdots & \ddots & \vdots \\ x_{N-m+1} & x_{N-m+2} & \cdots & x_{N} \end{array}\right]$$

Let $d_{ij}$ be the maximum distance between two templates *i* and *j* and *r* be a predetermined threshold.

(2) $$d_{ij}=\max\left\{|x\left(i+k\right)-x\left(j+k\right)|:0\leq k\leq m-1\right\}$$

Next, the number of similarities $n_{i}^{m}\left(r\right)$ between templates $u_{m}\left(i\right)$ and $u_{m}\left(j\right)$ can be calculated as follows:

(3) $$n_{i}^{m}\left(r\right)=\sum_{j=1}^{N-m}d\left[u_{m}\left(i\right),u_{m}\left(j\right)\right]$$

where similarity is defined as follows:

(4) $$d\left[u_{m}\left(i\right),u_{m}\left(j\right)\right]=\left\{\begin{array}{cc} 1 & d_{ij}\leq r\\ 0 & d_{ij}>r \end{array}\right.$$

The distance between templates is calculated through Equation (2) and then substituted into Equation (4) to define the similarity between the two. The two templates are determined to be similar when the distance $d_{ij}$ does not exceed the threshold *r*. By contrast, the two templates are dissimilar when $d_{ij}$ exceeds *r*. Different pattern templates can be substituted for comparisons with template *i*. The degree of sample similarity $U_{i}^{m}\left(r\right)$ can then be calculated as follows:

(5) $$U_{i}^{m}\left(r\right)=\frac{n_{i}^{m}\left(r\right)}{\left(N-m+1\right)}$$

The average degree of sample similarity can be calculated as follows after obtaining the degree of sample similarity:

(6) $$U^{m}\left(r\right)=\frac{1}{N-m}\sum_{i=1}^{N-m}U_{i}^{m}\left(r\right)$$

Here, $U^{m}\left(r\right)$ represents the average degree of similarity between all templates of length *m* in the template space *T*. Subsequently, a new template space is created by assembling templates with length *m* + 1. The average degree of similarity $U^{m+1}\left(r\right)$ of the new template space is calculated by repeating the aforementioned steps. Consequently, the SampEn values of the time series with parameters *m*, *r* and *N* can be obtained as follows:

(7) $$S_{E}\left(m,r,N\right)=-\ln\frac{U^{m+1}\left(r\right)}{U^{m}\left(r\right)}$$

### 2.2. MSE

MSE can extract much more information of a time series, compared with single scale-based entropy methods. Briefly, a time series is subjected to a coarse-graining procedure to construct multiple time series at different time scales [15,16]. The procedure is described as follows: A discrete time series $x_{1},x_{2},\cdots ,x_{N}$ with length *N* is segmented into multiple time series with length τ, where τ(τ=1,2,⋯,N) is the scale factor. Subsequently, a new time series $\left\{y_{j}^{\left(\tau \right)}\right\}$ is constructed by deriving the arithmetic mean of each set of data values according to the following equation:

(8) $$y_{j}^{\left(\tau \right)}=\frac{1}{\tau }\sum_{i=(j-1)\tau +1}^{j\tau }x_{i},1\leq j\leq \frac{N}{\tau }$$

The length of each coarse-grained time series is N/τ, meaning that at scale 1, the coarse-grained time series is the original time series. Moreover, the length of the coarse-grained time-series decreases as τ increases. After the process is completed, SampEn is used to calculate the entropy values for each coarse-grained time series $\left\{y_{j}^{\left(\tau \right)}\right\}$. The obtained sample entropy values are the MSE of the time series and are plotted as a function of the scale factor $\left(f\left(\tau \right)=S_{E}\right)$.

### 2.3. Cross-SampEn

With a similar procedure to SampEn, Cross-SampEn is used to evaluate the degree of dissimilarity between two time series derived from the same system [20]. The estimation of Cross-SampEn can be summarized as follows. Consider two individual time series $\left\{X_{i}\right\}=\left\{x_{1},\cdots ,x_{i},\cdots ,x_{N}\right\}$ and $\left\{Y_{j}\right\}=\left\{y_{1},\cdots ,y_{j},\cdots ,y_{N}\right\}$ with length *N*. The signals are segmented into the following templates of length *m*: $u_{m}\left(i\right)=\left\{x_{i},x_{i+1},\cdots ,x_{i+m-1}\right\},1\leq i\leq N-m+1$ and $v_{m}\left(j\right)=\left\{y_{j},y_{j+1},\cdots ,y_{j+m-1}\right\},1\leq j\leq N-m+1$. The template space $T_{x}$ is presented as follows:

(9) $$T_{x}=\left[\begin{array}{cccc} x_{1} & x_{2} & \cdots & x_{m}\\ x_{2} & x_{3} & \cdots & x_{m-1}\\ \vdots & \vdots & \ddots & \vdots \\ x_{N-m+1} & x_{N-m+2} & \cdots & x_{N} \end{array}\right]$$

Similarly, template space $T_{y}$ is expressed as follows:

(10) $$T_{y}=\left[\begin{array}{cccc} y_{1} & y_{2} & \cdots & y_{m}\\ y_{2} & y_{3} & \cdots & y_{m-1}\\ \vdots & \vdots & \ddots & \vdots \\ y_{N-m+1} & y_{N-m+2} & \cdots & y_{N} \end{array}\right]$$

The number of similarities between $u_{m}\left(i\right)$ and $v_{m}\left(j\right)$, defined as $n_{i}^{m}\left(r\right)$, is calculated under the following criterion:

(11) $$d[u_{m}\left(i\right),v_{m}\left(j\right)]\leq r,1\leq j\leq N-m$$

The similarity probability of the templates is evaluated using the following equations:

(12) $$U_{i}^{m}\left(r\right)\left(v∥u\right)=\frac{n^{m}\left(r\right)}{\left(N-m\right)}$$

Then, the average probability of similarity of length *m* is calculated as follows:

(13) $$U^{m}\left(r\right)\left(v∥u\right)=\frac{1}{\left(N-m\right)}\sum_{i=1}^{N-m}U_{i}^{m}\left(r\right)\left(v∥u\right)$$

where $U^{m}\left(r\right)\left(v∥u\right)$ is the degree of dissimilarity between the two time series when *m* points are segmented. Subsequently, new template spaces $T_{x}$ and $T_{y}$ are created by assembling templates with length m+1. The steps above are repeated to obtain the average similarity probability $U^{m+1}\left(r\right)\left(v∥u\right)$, and the Cross-SampEn values can be then derived as follows:

(14) $$CS_{E}\left(m,r,N\right)=-\ln\frac{U^{m+1}\left(r\right)\left(v∥u\right)}{U^{m}\left(r\right)\left(v∥u\right)}$$

### 2.4. MSCE and DI

The SHM system proposed in this study relies on MSCE analysis for identifying structural damage. Because an extended three-bay model was analyzed in this study, it was also of interest to identify the damaged bay. However, diagnosing the location of damage through a simple observation of the obtained MSCE curves is typically difficult. Therefore, a DI based on [22,23] was proposed for rapidly and efficiently diagnosing the damaged floor, axis and bay in the structure.

For the three-bay structure described in the following section, the SHM process is applied to each bay separately to detect the possible damage, while the interaction between each bay has been included in the measured signals. Thus, the following procedure is repeated three times. Two groups of curves representing the healthy and damaged conditions of the structure are analyzed. For a biaxial structure with *N* floors, the MSCE curves for the *x*- and *y*-axes under the healthy condition can be expressed as matrices:

(15) $$MSCE_{undamaged}=\left\{\begin{array}{c} H_{1}^{x}\\ H_{2}^{x}\\ \vdots \\ H_{N}^{x} \end{array}\right\}\;MSCE_{undamaged}\left\{\begin{array}{c} H_{1}^{y}\\ H_{2}^{y}\\ \vdots \\ H_{N}^{y} \end{array}\right\}$$

Similarly, the MSCE curves for the *x*- and *y*-axes under the damaged condition are expressed as follows:

(16) $$MSCE_{damaged}=\left\{\begin{array}{c} D_{1}^{x}\\ D_{2}^{x}\\ \vdots \\ D_{N}^{x} \end{array}\right\}\;MSCE_{damaged}\left\{\begin{array}{c} D_{1}^{y}\\ D_{2}^{y}\\ \vdots \\ D_{N}^{y} \end{array}\right\}$$

*H* and *D* represent the MSCE curves for the healthy and damaged conditions, respectively. The superscripts *x* and *y* represent the analyzed axes, and the subscript depicts the analyzed floor. For example, $H_{N}^{x}$ is the *x*-axis MSCE of the signal in the analyzed *N*-th floor and the signal of the floor beneath it under healthy conditions. This can be further expressed as follows:

(17) $$H_{N}^{axis}=\left\{CS_{E\;HNaxis}^{\;\;1},CS_{E\;HNaxis}^{\;\;2},CS_{E\;HNaxis}^{\;\;3},\cdots ,CS_{E\;HNaxis}^{\;\;\tau }\right\}$$

Similarly, $D_{N}^{axis}$ can be arranged as follows:

(18) $$D_{N}^{axis}=\left\{CS_{E\;DNaxis}^{\;\;1},CS_{E\;DNaxis}^{\;\;2},CS_{E\;DNaxis}^{\;\;3},\cdots ,CS_{E\;DNaxis}^{\;\;\tau }\right\}$$

where $CS_{E}$ denotes the Cross-SampEn value in each element, the superscript denotes the scale factor τ, the first superscript denotes the health condition, the second subscript denotes the analyzed floor and the third subscript denotes the analyzed axis. Subsequently, the following formulae can be used to calculate the dual-axis DI per floor on a specific bay:

(19) $$DI_{Nx}=\sum_{k=1}^{\tau }\left(CS_{E\;DNx}^{\;\;q}-CS_{E\;HNx}^{\;\;q}\right)\;\;DI_{Ny}=\sum_{k=1}^{\tau }\left(CS_{E\;DNy}^{\;\;q}-CS_{E\;HNy}^{\;\;q}\right)$$

The DI is evaluated by calculating the difference between the MSCE values of the damaged and healthy structures. Each bay of the structure has two DI values per floor: one on the *x*-axis and the other on the *y*-axis. For a specific floor, a positive DI indicates that the floor has sustained damage, whereas a negative DI indicates no damage on the floor, where the structure is under a more stable condition compared to the original healthy state. As verified in previous research [22], the proposed method can sustain the velocity changes for an approximately 10–20% noise level.

## 3. Numerical Simulation

To verify the feasibility of the SHM system proposed in this study, SAP2000 software (SAP2000 v9) was used to construct and analyze the three-bay, seven-story model, which is an extension of the areal benchmark structure commonly used at the National Center for Research on Earthquake Engineering (NCREE), for numerical simulation. The dimensions and characteristics of the numerical model are outlined as follows: The model was a steel structure comprising seven stories and three bays on the *x*-axis and a single bay on the *y*-axis. The height of each story was 1.06 m, and the widths of the bays on the *x*- and *y*-axes were 1.32 and 0.92 m, respectively. The columns were steel plates measuring 75×50 ${\mathrm{mm}}^{2}$. The beams were steel plates of 70×100 ${\mathrm{mm}}^{2}$. All sides of the structure were fitted with steel bracing chosen as L-shaped steel angles measuring 65×65×6 mm. Apart from the self-weight of the structure, an additional 500-kg mass was added per bay on each story to simulate the actual characteristics of a structure.

**Figure 1:**
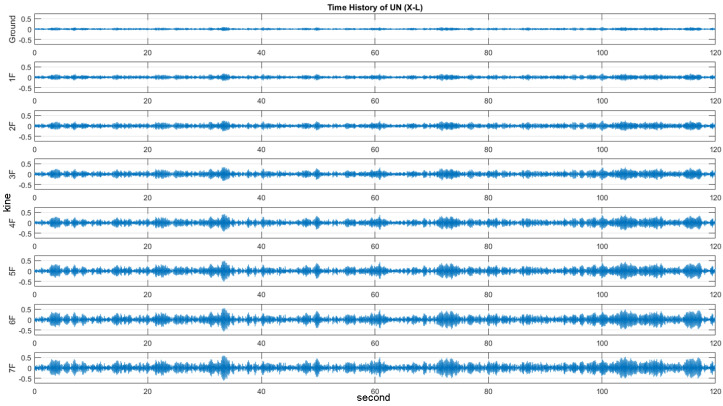
The time history of the velocity response of the undamaged scenario.

Time history and modal analyses were performed on the model. To perform the time history analysis, white noise signals were first generated with a power intensity of 1 MW and selected as the input accelerations to excite the numerical model as a simulation of ambient vibrations. The direct integration time-history analysis was applied to solve the structural response for 150 s. The response signals of the undamaged scenario under ambient vibrations shown in Figure 1 were used as the reference of the SHM database for damage detection. Detailed setting of the finite element model is listed in Table 1.

### Damage Database

The numerical model of the steel structure was fitted with diagonal braces on all directions of every floor, implying that for the three-bay model, each floor had six braces on the strong (X) axis and four braces on the weak (Y) axis. Structural damage was simulated by removing the installed braces symmetrically; that is, two braces were removed on the *x*-axis to simulate damage on the strong axis, and two bracings were removed on the *y*-axis to simulate damage on the weak axis.

**Figure 2:**
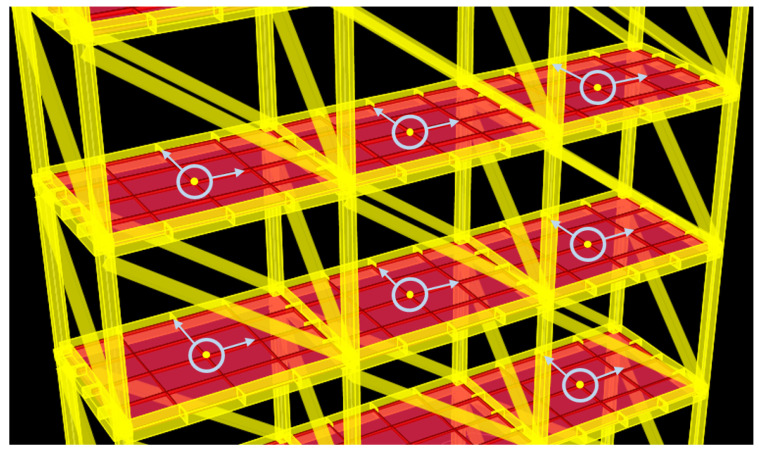
Signal extraction points.

**Figure 3:**
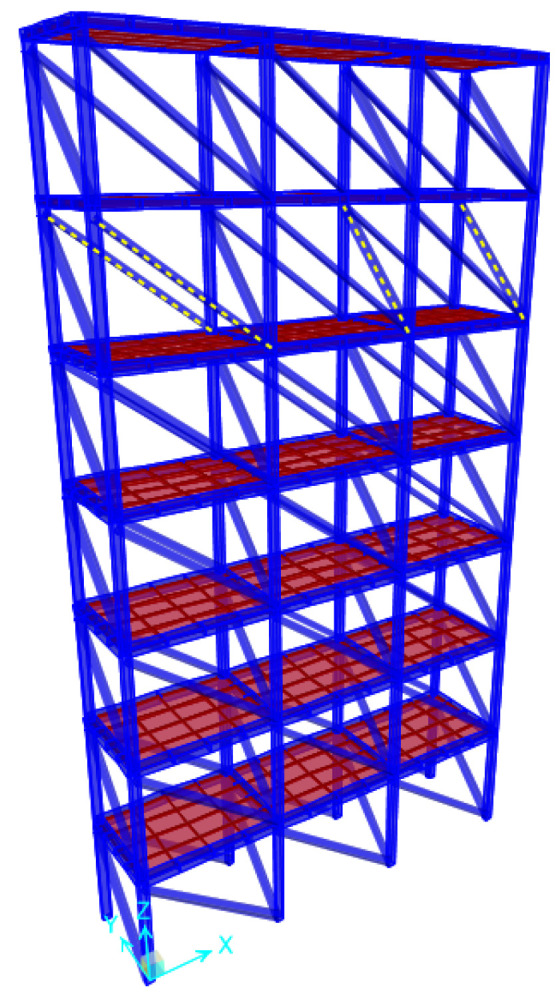
Three-dimensional view of the numerical model. The dotted braces represent the damaged bracing for Case 9 (6X-L & 6Y-R).

After the SHM database was constructed by performing the time history and modal analyses on the numerical model for each damage condition, biaxial velocity response data were extracted from the center of each floor per bay. Figure 2 shows the data extraction points of the structure. The various damage conditions along with their respective cases are presented in Table 2. The cases comprised various combinations of single-story, two-story or multistory damage, paired with single- or multi-bay and single- or multi-direction damage. The scenarios for the damage database were selected with a broad spectrum of diverse damage locations. In total, the damage conditions were classified into 12 categories and 26 cases. Numbers in the case names indicate damaged floors; X and Y represent the damaged axes; and L, C and R (left, center and right) denote the damaged bay. For example, Case 9 (6X-L & 6Y-R) represents a case involving damage on the sixth floor, *x*-axis, left bay and sixth floor, *y*-axis, right bay. Figure 3 illustrates the numerical model, with the dotted lines representing the braces removed to exemplify the damaged bracing for Case 9. A list of the modal analysis for all damage cases is shown in Table 3 to reflect the change of the global behavior of the numerical model. As depicted, the fundamental frequencies of the first and second modes drop accompanying the increase of damage level in both the X and Y directions. Significant changes are caused by damages located in the lower floors.

## 4. Results and Discussion

For the undamaged case and every damage condition, the velocity signals for the long (X) and short (Y) axes were extracted per bay from the center of each floor. Subsequently, the signals of two vertically-adjacent floors under the same damage condition were processed through Cross-SampEn at multiple scales (MSCE) to evaluate the dissimilarity between floors. After a series of optimization searches [23], where different combinations of parameters were considered for the best performance on damage detection accuracy, the parameters required for the calculation of Cross-SampEn, such as the template length *m*, threshold *r* and signal length *N*, were selected as 4, 0.10× the standard deviation (SD) of the time series and 30,000, respectively. In addition, Cross-SampEn was calculated across 25 scales (τ = 25).

After analyzing the undamaged case and the 26 damage cases through the MSCE method, the results were compared and quantified per bay using the DI. When a specific floor sustains damage, the structure experiences a loss of stiffness, which causes a rise in the complexity of the extracted response signals for that specific floor. Time series with high complexity have high entropy values, indicating damage. Thus, analysis of the changes in complexity or entropy of healthy and damaged signals results in positive DI values for damaged floors and negative DI values for healthy floors. Small positive values can be excluded by a predetermined threshold value of one, which was selected by experimentation. Thus, the damaged floor and direction can be easily detected.

**Figure 4:**
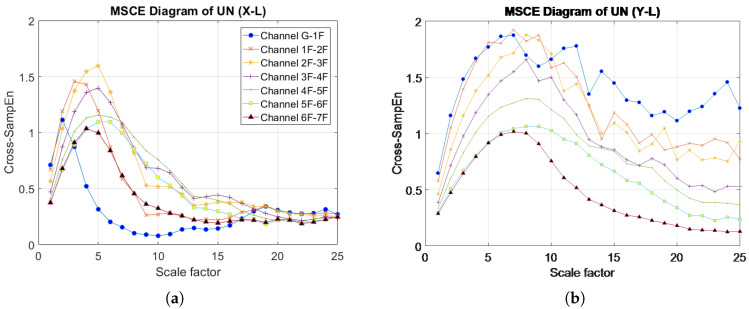

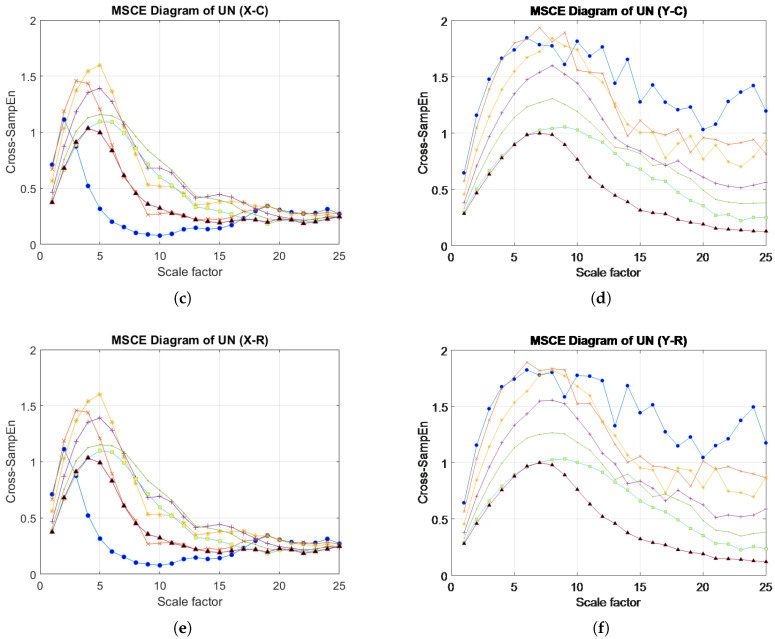
MSCE diagrams for undamaged condition: (**a**) left bay, X-direction; (**b**) left bay, Y-direction; (**c**) center bay, X-direction; (**d**) center bay, Y-direction; (**e**) right bay, X-direction; (**f**) right bay, Y-direction.

Figure 4a–f presents the MSCE diagrams obtained for the healthy condition in the X- and Y-directions for the left, center and right bays; the diagrams of the X-direction are shown on the left side, and those of the Y-direction are on the right side. In these figures, G-1F denotes the curve for the first floor, 1F-2F denotes the curve for the second floor, 2F-3F denotes the curve for the third floor, and so forth. The Cross-SampEn for Channel G-1F was calculated from the velocity signals of the ground and the first floor.

### 4.1. Single-Story, Single-Bay, Multidirectional Damage: Case 4 (4XY-L)

**Figure 5:**
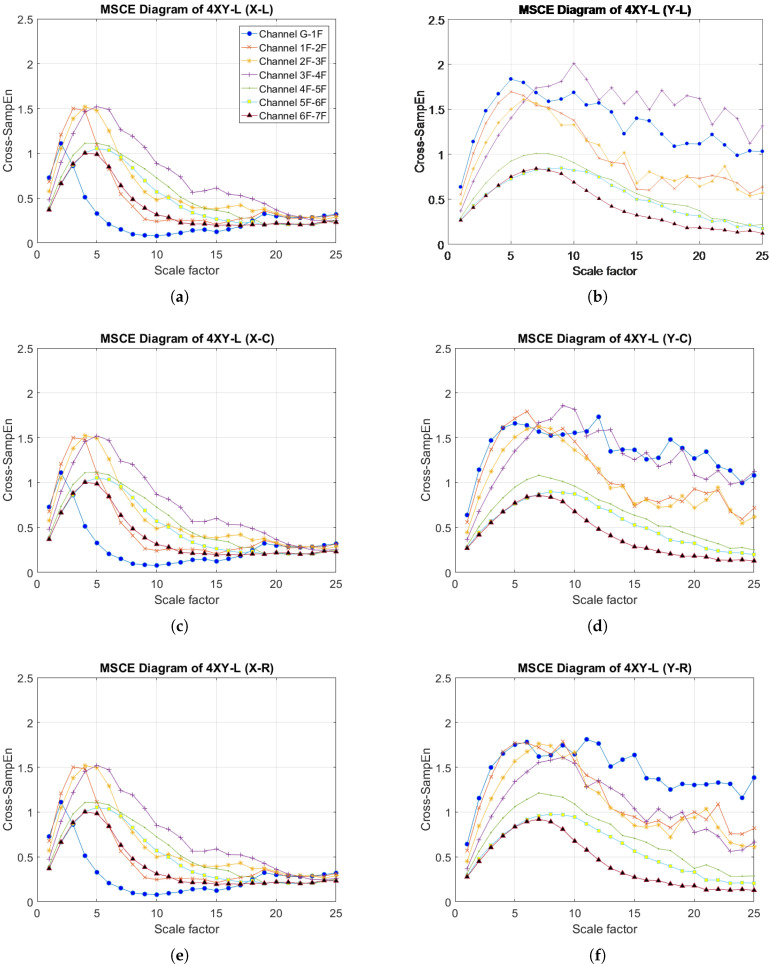
MSCE diagrams for Case 4: damage on fourth floor, left bay, X- and Y-directions: (**a**) left bay, X-direction; (**b**) left bay, Y-direction; (**c**) center bay, X-direction; (**d**) center bay, Y-direction; (**e**) right bay, X-direction; (**f**) right bay, Y-direction.

Figure 5 illustrates the MSCE diagrams for Case 4. In the X-direction, the rise of the curve for the fourth floor is evident in the MSCE diagrams of all bays, whereas the remaining curves are in similar positions as in the healthy condition. In the Y-direction, the rise of the fourth-floor curve and the dropping of the remaining curves are also noticeable in the diagrams of all bays. Furthermore, Figure 5b shows a large gap between the fourth-floor curve and the remaining curves at scale factors ranging from 8–25. These results indicate damage on the fourth floor in both directions, with a higher degree of damage possibly occurring on the left bay.

**Figure 6:**
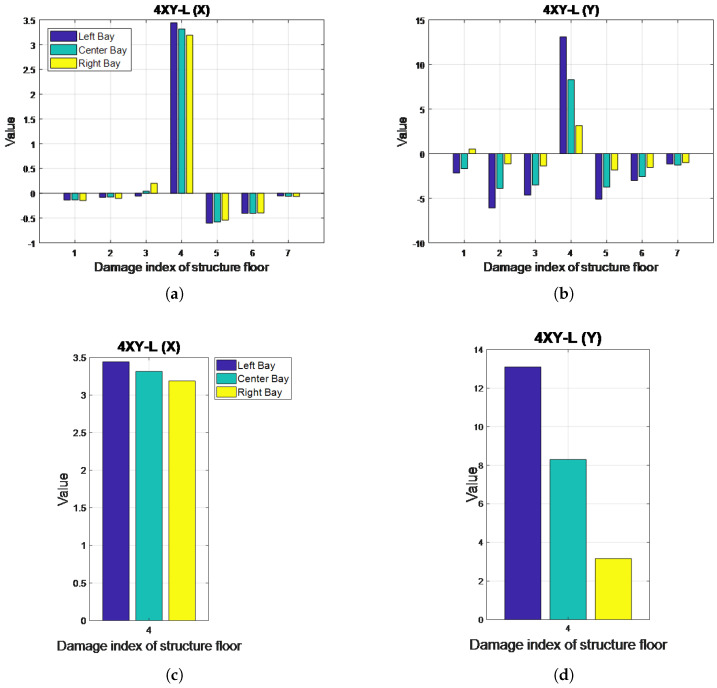
Damage index (DI) diagrams for Case 4: (**a**) X-direction; (**b**) Y-direction; (**c**) close up of DI diagrams for the fourth floor in Case 4: X direction; (**d**) Y-direction.

The DI analysis results shown in Figure 6 indicate that the fourth floor sustained damage in the X- and Y-directions. Figure 6c,d enables a closer evaluation of the DI values of the fourth floor. In both directions, the bar representing the left bay is higher than the remaining bars, indicating that this bay could have sustained more damage. In the Y-direction, the DI values are shown to be higher, and the differences among bays are shown to be more significant than those in the X-direction. Because the analyzed model involved three bays in the X-direction and a single bay in the Y-direction, the removal of bracings in the short (Y) axis could foster a larger impact on its stiffness; therefore, more significant differences could be observed.

### 4.2. Single-Story, Multi-Bay, Multidirectional Damage: Case 8 (3X-R & 3Y-C)

**Figure 7:**
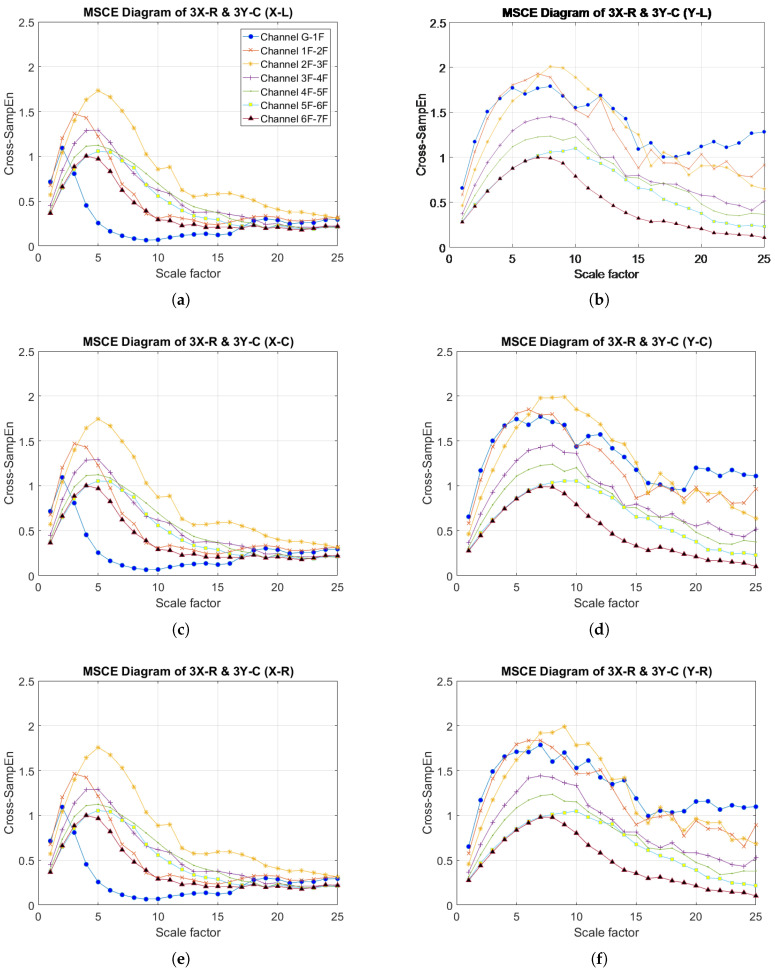
MSCE diagrams for Case 8: damage on the third floor, right bay, X-direction and third floor, center bay, Y-direction: (**a**) left bay, X-direction; (**b**) left bay, Y-direction; (**c**) center bay, X-direction; (**d**) center bay, Y-direction; (**e**) right bay, X-direction; (**f**) right bay, Y-direction.

The results for the MSCE analysis of Case 8 are shown in Figure 7. In the X-direction, the MSCE diagrams for the three bays are very similar to one another. The figure reveals that the curves for the third floor are in higher positions than they are in the diagram for the healthy condition. Furthermore, at scales ranging from 4–24, the third-floor curves are shown to maintain significantly higher positions than the remaining curves. In the Y-direction, the curves for the third floor are also shown to rise relative to their original positions in the diagram for the undamaged condition, with the difference being slightly higher for the MSCE diagram for the center bay (Figure 7d). Case 8 was therefore classified as involving damage in both directions of the third floor.

**Figure 8:**
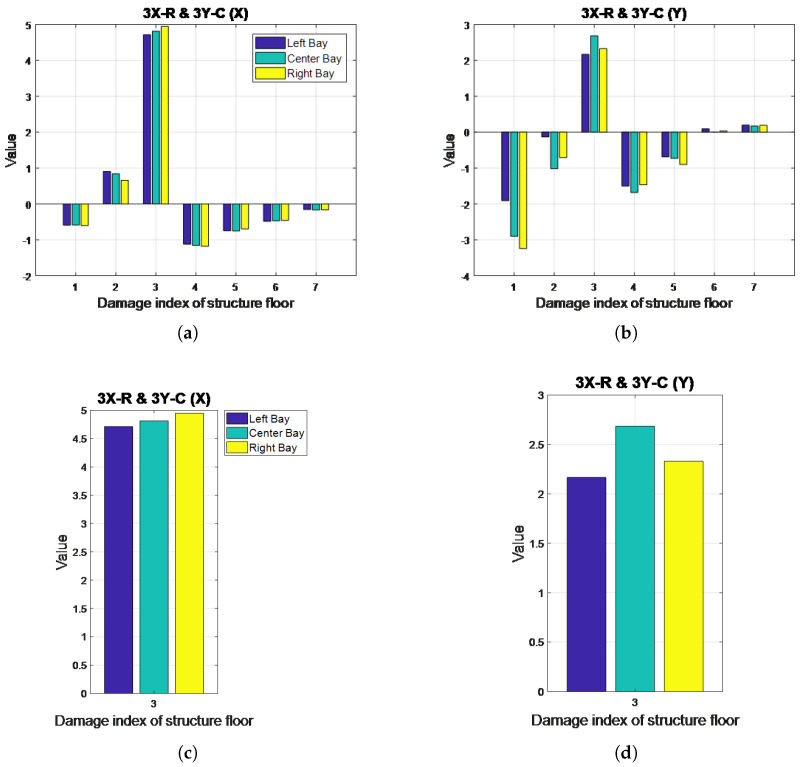
DI diagrams for Case 8: (**a**) X-direction; (**b**) Y-direction; close-up of DI diagrams for the third floor in Case 8: (**c**) X-direction; (**d**) Y-direction.

The DI diagrams for Case 8 are illustrated in Figure 8. In the X-direction, a positive DI value is shown for the third floor, with the right bay bar revealing the highest DI. Although the second floor also had a positive DI, it was excluded because the value fell under the threshold. Because damage happened on the third floor, an effect on the adjacent second floor is natural due to the production of a rigid body response. The DI diagram for the Y-direction also reveals a positive DI for the third floor. In this case, the center bay bar is shown to be the highest. These results, which can be examined more closely in Figure 8c,d, confirm the MSCE analysis findings, with a further indication of the possible damaged bay.

### 4.3. Two-Story, Single-Bay, Single-Direction Damage: Case 11 (1Y-R & 5Y-R)

**Figure 9:**
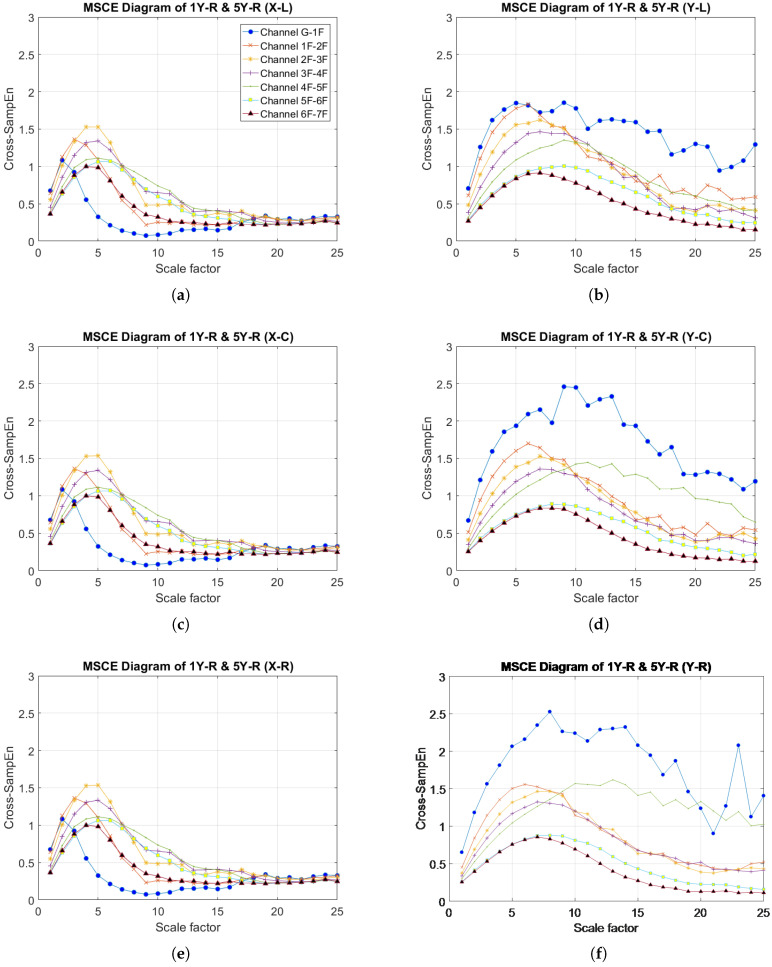
MSCE diagrams for Case 11: damage on first floor, right bay, Y-direction and fifth floor, right bay, Y-direction: (**a**) left bay, X-direction; (**b**) left bay, Y-direction; (**c**) center bay, X-direction; (**d**) center bay, Y-direction; (**e**) right bay, X-direction; (**f**) right bay, Y-direction.

The results obtained from the MSCE analysis of Case 11 are presented in Figure 9. In the X-direction, the MSCE curves for all bays remain almost identical to those of the undamaged condition; therefore, no damage occurred in the X-direction. Figure 9b illustrates the MSCE curves for the left bay in the Y-direction, revealing that that the curve for the first floor is at a higher position than the remaining curves at most scales. Furthermore, at scales of 5–15, a rise in complexity can be observed for the fifth floor. The changes in the curves for the first and fifth floors are even more evident in the MSCE diagrams for the center and right bays (Figure 9d,f). Therefore, the first and fifth floors were damaged in the Y-direction. Additionally, the curve for the fifth floor in the right bay displays higher entropy values than in the center bay for scales ranging from 10–25, suggesting a higher degree of damage to the right bay.

**Figure 10:**
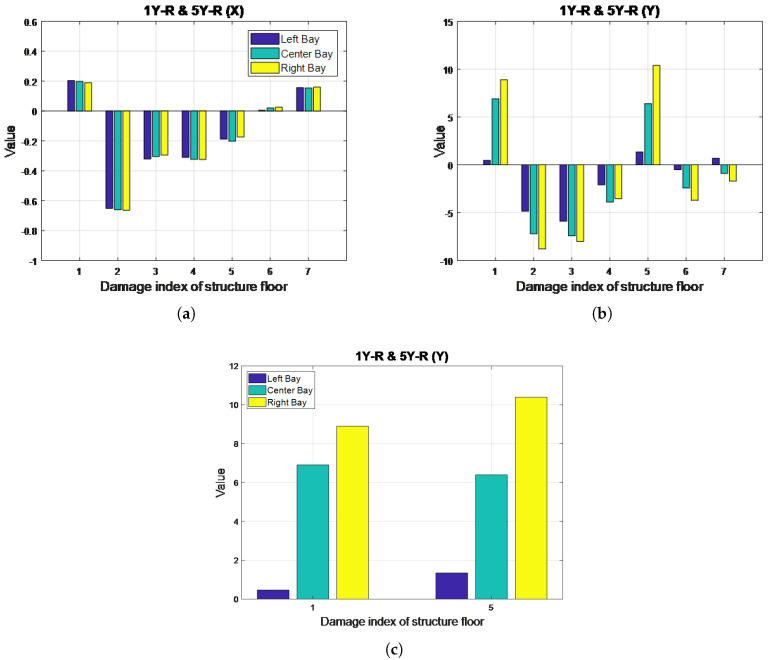
DI diagrams for Case 11: (**a**) X-direction; (**b**) Y-direction; (**c**) close-up of DI diagrams for the first and fifth floors in Case 11 (Y-direction).

The DI analysis results are shown in Figure 10, indicating no damage in the X-direction. Moreover, in the Y-direction, the first and fifth floors are demonstrated to have positive DI values. Figure 10c shows a close-up of the DI values obtained for the first and fifth floors in the Y-direction; the bars representing the right bay exhibit the highest values. Therefore, the first and fifth floors were damaged in the Y-direction, with the right bay sustaining a higher degree of damage. Hence, the MSCE analysis results are confirmed for this case.

### 4.4. Two-Story, Multi-Bay, Single-Direction Damage: Case 16 (2X-L & C, 6X-C & R)

**Figure 11:**
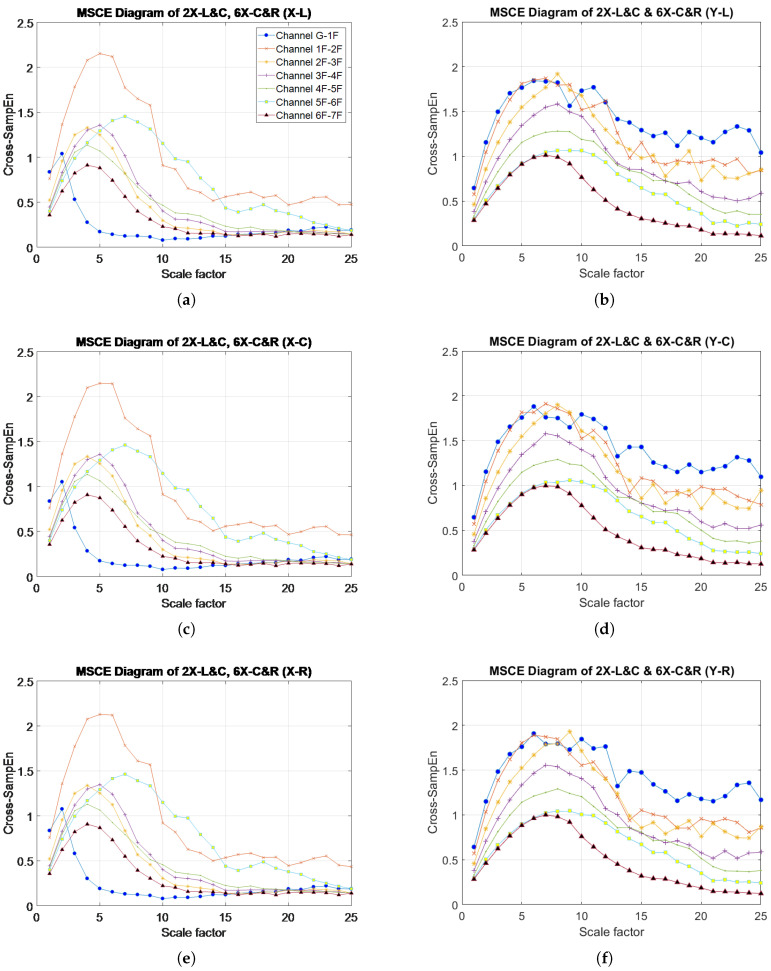
MSCE diagrams for Case 16: damage on second floor, left and center bays, X-direction and sixth floor, center and right bays, X-direction: (**a**) left bay, X-direction; (**b**) left bay, Y-direction; (**c**) center bay, X-direction; (**d**) center bay, Y-direction; (**e**) right bay, X-direction; (**f**) right bay, Y-direction.

Figure 11 illustrates the MSCE curves obtained from the analysis of Case 16. The curves for the second and sixth floors are shown to clearly separate and maintain a wide gap from the remaining curves in the MSCE diagrams obtained from analysis of the three bays in the X-direction. The curves for the second and sixth-floor reach their peaks at scales 5 and 7, respectively. In the Y-direction, no evident changes are shown in the diagrams for any bay; therefore, damage was determined to have occurred on only the second and sixth floor in the X-direction.

**Figure 12:**
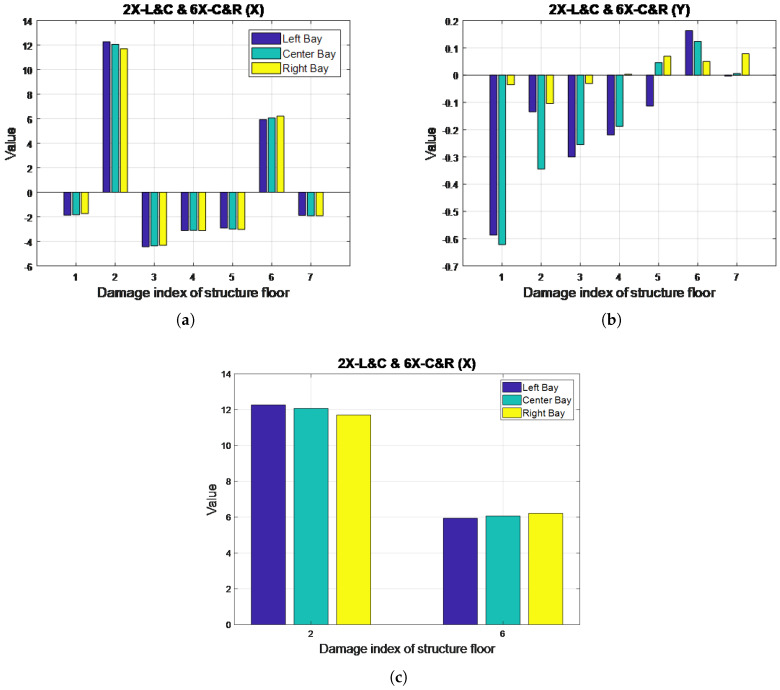
DI diagrams for Case 16: (**a**) X-direction; (**b**) Y-direction; (**c**) close-up of DI diagrams for the second and sixth floors in Case 16 (X-direction).

The DI diagrams for Case 16 are shown in Figure 12. In the X-direction, positive DI values are shown for the second and sixth floors, thus resulting in these floors being classified as damaged. The DI value of the second floor is higher than that in the sixth floor because the damage on the second floor can relatively affect the global behavior of the structure compared with similar damage conditions. In this particular case, the second floor was damaged on the left and center bays, whereas the sixth floor was damaged on the center and right bays. The damaged bay could not be identified by the DI during the analysis of damage to a single floor in adjacent bays; however, the results could still indicate that the second and sixth floors received more damage in the left and right bays, respectively. Figure 12c enables a closer observation of these results. Finally, the DI diagram for the Y-direction indicates no damage on this axis.

### 4.5. Multistory, Multi-Bay, Single-Direction Damage: Case 23 (3X-L & 4X-C & 5X-R)

**Figure 13:**
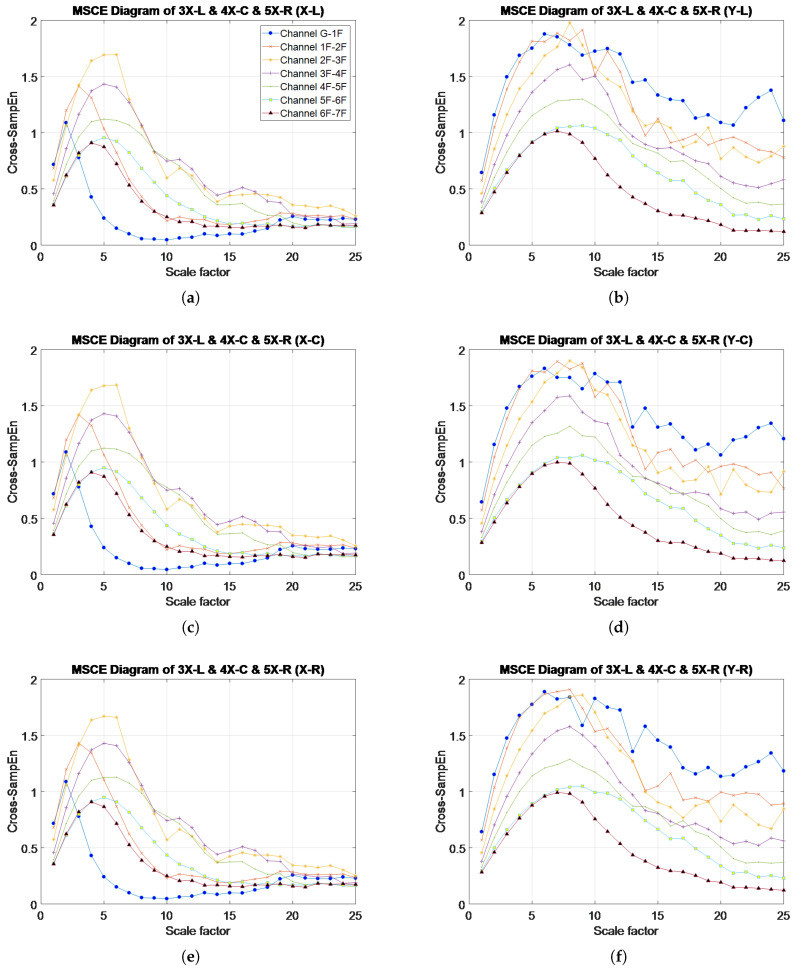
MSCE diagrams for Case 23: damage on the third floor, left bay, X-direction; fourth floor, center bay, X-direction; fifth floor, right bay, X-direction: (**a**) left bay, X-direction; (**b**) left bay, Y-direction; (**c**) center bay, X-direction; (**d**) center bay, Y-direction; (**e**) right bay, X-direction; (**f**) right bay, Y-direction.

The MSCE diagrams for Case 23 are illustrated in Figure 13. For the three bays in the X-direction, an increase in complexity is shown for the third and fourth floors. The fifth-floor curve is shown to remain in an almost identical position as in the diagram for the healthy condition, whereas the remaining curves are illustrated to decrease relative to their positions under the healthy condition. Although the fifth floor was incorrectly classified as undamaged, the third and fourth floors were determined as damaged in the X-direction. To determine the damaged bays, further examination was required through DI analysis. No evident changes were observed in the Y-direction.

**Figure 14:**
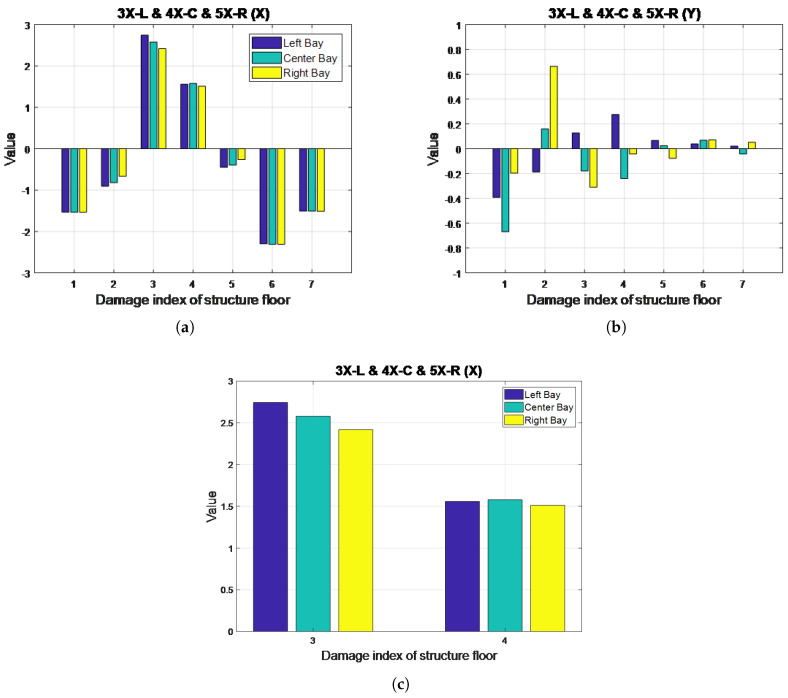
DI diagrams for Case 23: (**a**) X-direction; (**b**) Y-direction; (**c**) close-up of DI diagrams for the third and fourth floors in Case 23 (X-direction).

The DI diagrams for this case are presented in Figure 14, revealing positive DI values for the third and fourth floors in the X-direction. The resulting diagram, a close-up of which is provided in Figure 14c, suggests that the third floor sustained more damage in the left bay, whereas the bar is slightly higher for the center bay on the fourth floor. The fifth floor was not identified as damaged. In the Y-direction, small positive DI values are shown in the figure; however, these values were determined to be negligible because they fell under the predetermined threshold of one, thereby confirming the MSCE analysis results.

### 4.6. Multistory, Multi-Bay, Multidirectional Damage: Case 26 (7XY-R & 4Y-L & 6Y-C)

**Figure 15:**
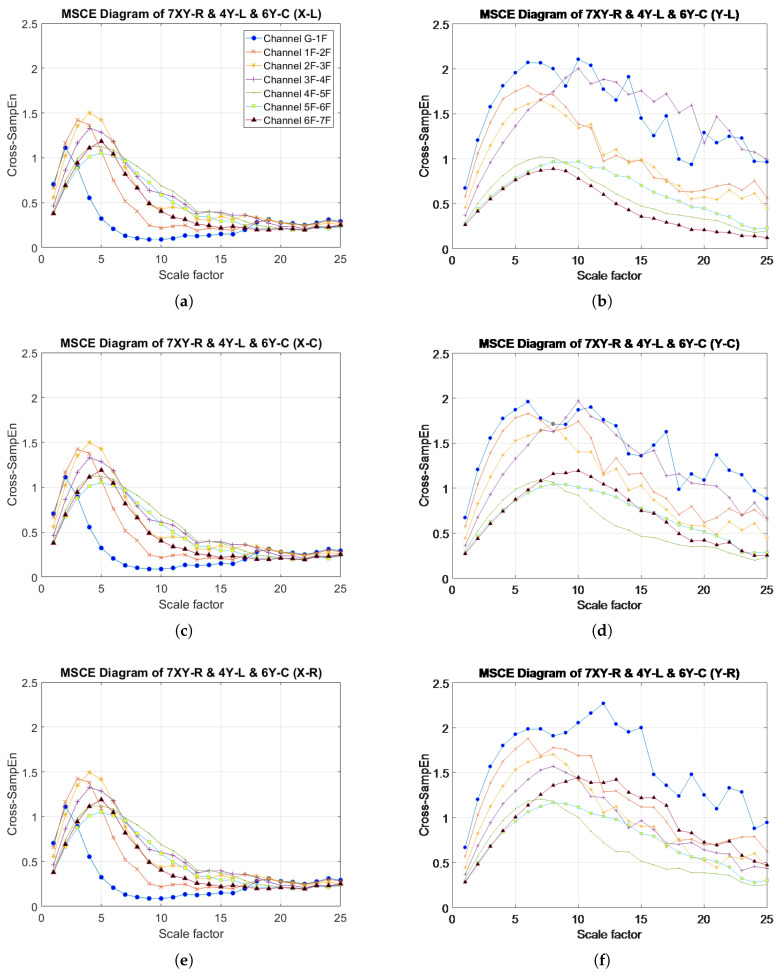
MSCE diagrams for Case 26: damage on seventh floor, right bay, X- and Y-directions; fourth floor, left bay, Y-direction; sixth floor, center bay, Y-direction: (**a**) left bay, X-direction; (**b**) left bay, Y-direction; (**c**) center bay, X-direction; (**d**) center bay, Y-direction; (**e**) right bay, X-direction; (**f**) right bay, Y-direction.

Figure 15 illustrates the MSCE curves for Case 26. In the X-direction, the curves for the seventh floor for all bays are shown to increase and reach their peaks at scale 5. However, the curves for the remaining floors are shown to drop or maintain their positions as in the diagrams for the healthy condition. Therefore, the seventh floor was determined to have sustained damage in the X-direction. In the Y-direction, the MSCE diagram for the left bay (Figure 15b), reveals that the curve for the fourth-floor increases dramatically. The trend for the first floor also increases, albeit slightly erratically. A slight increase in the curves for the sixth and seventh floors can be observed in the MSCE diagram for the center bay (Figure 15d). Moreover, a change is observed in the fourth-floor curve, but to a lower degree than that in the left bay MSCE diagram for the left bay. Regarding the MSCE diagram for the right bay (Figure 15f), the curves for the first, sixth and seventh floors increase significantly. On the basis of these results, the first, fourth, sixth and seventh floors sustained damage; however, this was false for the first floor.

**Figure 16:**
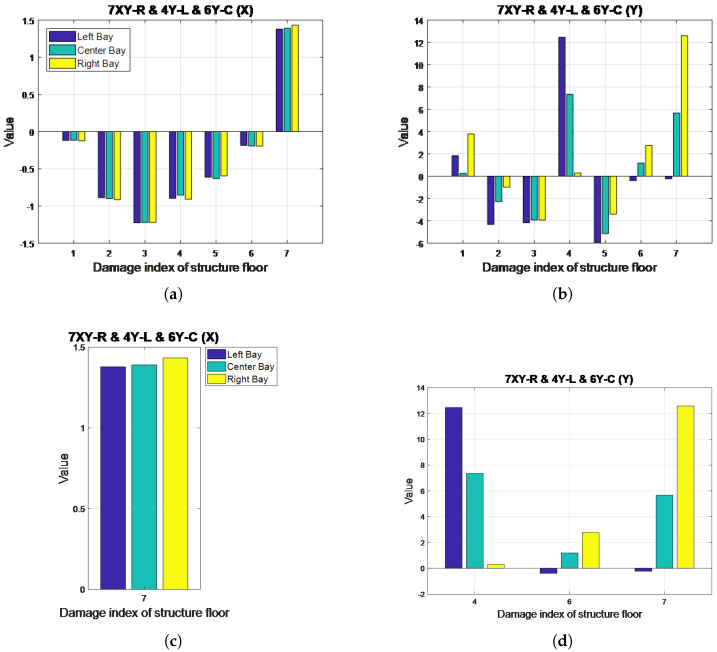
DI diagrams for Case 26: (**a**) X-direction; (**b**) Y-direction; (**c**) seventh floor, X-direction; (**d**) fourth, sixth and seventh floor, Y-direction.

Figure 16 illustrates the DI diagrams obtained for this case, with a close-up presented in Figure 16c,d. Positive DI values can be observed for the seventh floor in the X-direction, with the bar representing the right bay having the highest DI value. The first, fourth, sixth and seventh floors were determined to have sustained damage in the Y-direction. However, slightly erratic curves are shown in the figure for the first floor, which may have caused its misclassification. The DI diagrams suggest that the fourth and seventh floors received more damage in the left and right bays, respectively. For the sixth floor, the bar representing the right bay indicates the highest value, possibly because the damage to the center bay also affected the right bay because the bracing was shared by both. The results obtained in the DI analysis for this case demonstrate the accuracy of the proposed system.

### 4.7. General Discussion

In this study, 26 damage cases classified into 12 categories were examined to verify the feasibility of MSCE and DI analyses for damage detection in a complex three-bay structure. The complete results are summarized in Table 4. The DI values are lower when there are more damaged floors as the structural complexity is redistributed after different damage conditions. These were further analyzed in two stages. First, the DI results obtained for the identification of the damaged floors and damage directions were quantified through a precision and recall analysis. For the precision and recall analyses, the DI results for the X- and Y-directions were first classified into four categories: true positives (TP), representing damaged floors that have been correctly identified; false positives (FP), representing floors that have been misclassified as damaged; true negatives (TN), representing undamaged floors that have been correctly classified; and false negatives (FN), representing damaged floors that have been misclassified as undamaged. Precision and recall can then be calculated as follows:

(20) $$\mathrm{Precision}=\frac{TP}{TP+FP}\;\;\mathrm{Recall}=\frac{TP}{TP+FN}$$

#### 4.7.1. Damage Location

High precision denotes few false positives, which represents the percentage of detecting the damage location correctly, and a high recall indicates few false negatives, which indicates the reliability of not missing the possible damage. The combined results for both directions are summarized in Table 5. The derived precision and recall were 83% and 87%, respectively. These results indicate that 83% of the floors and their respective directions classified by the DI as damaged were true positive. As most of the damages were simulated along the X-direction, better performance was achieved in the X-direction for 100% than the Y-direction, which is 73% in the study. Moreover, 87% of all actual damaged floors and their respective directions were correctly classified as damaged. The high-percentage of accuracy proves that the system can reliably detect any level of damage with only a small probability of misdiagnoses, which are false negatives. The results have demonstrated the capacity of the proposed SHM system to locate the damaged floor and direction of a large, three-bay numerical model.

#### 4.7.2. Damaged Bay Identification

A separate analysis of the identification accuracy of the damaged bays was then performed; therefore, the results pertaining to the damaged bays were not considered for the precision and recall analyses. Because the removal of bracings on a specific bay would inevitably affect those adjacent to it, the calculated DI values would be fairly close. The combined results for both directions are shown in Table 6, indicating an average identification accuracy of 75%. Better performance was achieved in the Y-direction, as it only has one bay in the Y-direction. For the complicated damage combinations in the X-direction, the accuracy dropped to 71%. Furthermore, the proposed system is limited when more than one bay is damaged. Therefore, these results are merely a suggestion as to which bay might have been affected more severely. With the support of the damaged bay identification, the proposed method can be easily extended to any complex structure.

## 5. Conclusions

In this study, the feasibility of detecting damage in a complex three-bay, seven-story numerical model by SHM methods was examined. Through the proposed SHM system, damage locations can be rapidly and effectively detected by only measuring the velocity response data of the model. The ambient vibrations from the center of each floor before and after damage occurs were first recorded, and subsequently, the complexity of the signals can be analyzed by the MSCE method. The reliability and viability of the proposed SHM system were examined through the numerical analysis of 26 damage cases in 12 categories representing several degrees of damage severity. The results of the analyses were examined in two stages. First, the results pertaining to the damaged floor and direction indicate that 83% of the floors and their respective directions were truly damaged. Furthermore, 87% of all the actually damaged floors and their respective directions were correctly classified as damaged by the DI. Subsequently, the identification of the damaged bays was analyzed, and an identification accuracy of 75% was obtained. Identification of the damaged bays through the proposed SHM system is limited, especially when multi-bay damage exists on a single floor.

The obtained results verify the feasibility and further potential of the proposed SHM system for the detection and localization of damage in large and complex structures.

## Figures and Tables

**Table 1:** Numerical setting of the finite element model.

Parameter	Setting
Constitutive equation	Linear elastic
Material	A36 steel
DOF of the foundation	Fixed in 6 DOF
Finite element for column, beam and bracing	Beam
Integration method	Direct-integration time-history analysis
Duration	150 s
Ambient vibration	White noise of 1 mW power

**Table 2:** Damage cases for analysis.

Case Number	Damage Group	Damaged Floor, Direction and Bay
1	Single-story, single-bay, single-direction	5X-L
2		3Y-C
3		7Y-R
4	Single-story, single-bay, multidirectional	4XY-L
5		6XY-C
6	Single-story, multi-bay, single-direction	2X-L & 2X-C
7		5Y-L & 5Y-C & 5Y-R
8	Single-story, multi-bay, multidirectional	3X-R & 3Y-C
9		6X-L & 6Y-R
10	Two-story, single-bay, single-direction	3X-L & 6X-L
11		1Y-R & 5Y-R
12	Two-story, single-bay, multidirectional	4X-C & 7Y-C
13		2XY-R & 3XY-R
14	Two-story, multi-bay, single-direction	5X-R & 7X-L
15		2Y-C & 4Y-R
16		2X-L & C, 6X-C & R
17	Two-story, multi-bay, multidirectional	4X-R & 2Y-L
18		6XY-R & 7XY-L
19	Multistory, single-bay, single-direction	3X-L & 4X-L & 6X-L
20		1Y-R & 4Y-R & 7Y-R
21	Multistory, single-bay, multidirectional	4X-L & 5Y-L & 6Y-L
22		1XY-C & 3XY-C & 5XY-C
23	Multistory, multi-bay, single-direction	3X-L & 4X-C & 5X-R
24		6Y-L & 2Y-C & 7Y-R
25	Multistory, multi-bay, multidirectional	1X-R & 2X-R & 1Y-L
26		7XY-R & 4Y-L & 6Y-C

**Table 3:** Modal analysis of the numerical model.

Case No.	Damage Case	Frequency (Hz)			
		Mode 1(X)	Mode 2(X)	Mode 1(Y)	Mode 2(Y)
	Undamaged (UN)	6.5	19.52	2.69	11.56
1	5X-L	6.41	18.84	2.69	11.56
2	3Y-C	6.50	19.53	2.65	11.55
3	7Y-R	6.50	17.97	2.68	11.02
4	4XY-L	6.12	18.69	2.60	11.32
5	6XY-C	6.44	18.58	2.68	11.15
6	2X-L & 2X-C	5.78	18.93	2.69	11.57
7	5Y-L & 5Y-C & 5Y-R	6.27	17.41	2.44	8.88
8	3X-R & 3Y-C	6.32	19.52	2.65	11.55
9	6X-L & 6Y-R	6.43	17.02	2.66	10.48
10	3X-L & 6X-L	6.29	18.81	2.69	11.57
11	1Y-R & 5Y-R	5.88	17.55	2.49	9.21
12	4X-C & 7Y-C	6.32	19.09	2.69	11.36
13	2XY-R & 3XY-R	5.41	18.23	2.39	10.86
14	5X-R & 7X-L	6.42	18.55	2.69	11.56
15	2Y-C & 4Y-R	6.3	19.16	2.55	11.02
16	2X-L & C, 6X-C & R	5.67	16.21	2.69	11.57
17	4X-R & 2Y-L	5.91	18.66	2.57	11.00
18	6XY-R & 7XY-L	6.43	15.50	2.66	10.19
19	3X-L & 4X-L & 6X-L	6.13	18.52	2.69	11.57
20	1Y-R & 4Y-R & 7Y-R	5.78	16.13	2.45	9.33
21	4X-L & 5Y-L & 6Y-L	6.29	17.56	2.59	9.96
22	1XY-C & 3XY-C & 5XY-C	5.92	18.07	2.57	10.54
23	3X-L & 4X-C & 5X-R	6.12	18.46	2.70	11.57
24	6Y-L & 2Y-C & 7Y-R	6.49	16.44	2.60	9.94
25	1X-R & 2X-R & 1Y-L	5.67	18.09	2.56	10.40
26	7XY-R & 4Y-L & 6Y-C	6.27	15.94	2.59	10.52

**Table 4:** Classification results of DI analysis.

Case Number	Damage Group	Damage Case	Damage Index (X-Direction)	Damage Index (Y-Direction)
1	Single-story,	5X-L	OK	OK
2	single-bay,	3Y-C	OK	OK
3	single-direction	7Y-R	OK	1F ${}^{1}$
4	Single-story, single-	4XY-L	OK	OK
5	bay, multi-directional	6XY-C	6F ${}^{2}$	OK
6	Single-story, multi-	2X-L & 2X-C	2F ${}^{2}$	OK
7	bay, single-direction	5Y-L & 5Y-C & 5Y-R	OK	5F ${}^{2}$
8	Single-story, multi-	3X-R & 3Y-C	OK	OK
9	bay, multidirectional	6X-L & 6Y-R	OK	1F ${}^{1}$
10	Two-story, single-bay,	3X-L & 6X-L	OK	OK
11	single-direction	1Y-R & 5Y-R	OK	OK
12	Two-story, single-bay,	4X-C & 7Y-C	OK	OK
13	multidirectional	2XY-R & 3XY-R	OK	$1F^{1}$
14	Two-story,	5X-R & 7X-L	OK	OK
15	multi-bay,	2Y-C & 4Y-R	OK	1F ${}^{1}$ & 2F ${}^{2}$
16	single-direction	2X-L & C, 6X-C & R	2F ${}^{2}$ & 6F ${}^{2}$	OK
17	Two-story, multi-	4X-R & 2Y-L	OK	1F ${}^{1}$
18	bay, multidirectional	6XY-R & 7XY-L	OK	1F ${}^{1}$& 2F ${}^{1}$
19	Multistory, single-	3X-L & 4X-L & 6X-L	6F ${}^{3}$	OK
20	bay, single-direction	1Y-R & 4Y-R & 7Y-R	OK	OK
21	Multistory, single-	4X-L & 5Y-L & 6Y-L	OK	1F ${}^{1}$ & 2F ${}^{1}$
22	bay, multidirectional	1XY-C & 3XY-C & 5XY-C	1F ${}^{3}$ & 5F ${}^{3}$	1F ${}^{3}$ & 3F ${}^{2}$
23	Multistory, multi-bay,	3X-L & 4X-C & 5X-R	5F ${}^{3}$	OK
24	single-direction	6Y-L & 2Y-C & 7Y-R	OK	1F ${}^{1}$ & 2F ${}^{3}$
25	Multistory, multi-bay,	1X-R & 2X-R & 1Y-L	1F ${}^{3}$ & 2F ${}^{3}$	OK
26	multidirectional	7XY-R & 4Y-L & 6Y-C	OK	1F ${}^{1}$ & 6F ${}^{2}$

^1^ Indicates that the floor has been misclassified as damaged; ^2^ indicates that the damaged bay has not been successfully identified; ^3^ indicates that the damaged floor has not been detected.

**Table 5:** Precision and recall analysis results (X- and Y-directions).

Direction	True Positives	False Positives	True Negatives	False Negatives	Precision	Recall
X	25	0	151	6	100%	81%
Y	30	11	139	2	73%	94%
Total	55	11	290	8	83%	87%

**Table 6:** Identification accuracy of damaged bays (X- and Y-directions).

Direction	Damage Instances	Correctly Identified Bay	Accuracy
X	34	24	71%
Y	34	27	79%
Total	68	51	75%
